# Spatiotemporal Associations Between Deforestation and Acute Myocardial Infarction Mortality in the Brazilian Amazon

**DOI:** 10.3390/ijerph23070857

**Published:** 2026-06-30

**Authors:** Ryan Menezes Brito, Afrânio Gonçalves Neto, Marcus Lucas S. A. A. Souza, Sanderson Gustavo Ferreira da Silva, Stheffany Costa Bezerra, Grazielly Aguiar Ribeiro, Gabriela Alves Sales, Diego Simeone, Aldemir B. Oliveira-Filho

**Affiliations:** 1 Afya Faculdade de Ciências Médicas, Bragança 68600-000, PA, Brazil; enfermeiro.ryanbrito@gmail.com (R.M.B.); afranioneto008@gmail.com (A.G.N.); lucas.allencar2@gmail.com (M.L.S.A.A.S.); gustavin.18112004@gmail.com (S.G.F.d.S.); stheffany1costa@gmail.com (S.C.B.); grazielly.aguiara@gmail.com (G.A.R.); diego.simeone@afya.com.br (D.S.); 2 Programa de Pós-Graduação em Biologia Ambiental, Instituto de Estudos Costeiros, Universidade Federal do Pará, Bragança 68600-000, PA, Brazil; gabrielasalesbio@gmail.com; 3 Grupo de Estudo e Pesquisa em Populações Vulneráveis, Instituto de Estudos Costeiros, Universidade Federal do Pará, Bragança 68600-000, PA, Brazil

**Keywords:** cardiometabolic vulnerability, Bayesian inference, territorial inequalities, environmental surveillance, risk persistence, anthropogenic pressure

## Abstract

**Highlights:**

**Public health relevance—How does this work relate to a public health issue?**

- Deforestation was associated with increased acute myocardial infarction risk across health regions of the Brazilian Amazon.
- Areas experiencing greater environmental degradation showed progressive spatial intensification and temporal persistence of cardiovascular risk.

**Public health significance—Why is this work of significance to public health?**

- The findings demonstrate that large-scale environmental degradation may act as a structural socioenvironmental determinant of cardiovascular health.
- The study expands current evidence by linking deforestation to the spatiotemporal dynamics of noncommunicable diseases in tropical regions.

**Public health implications—What are the key implications or messages for practitioners, policy makers and/or researchers in public health?**

- Integrating environmental surveillance with cardiovascular health monitoring may improve identification of vulnerable territories and priority populations.
- Policies aimed at reducing deforestation may produce indirect cardiovascular health benefits by mitigating thermal stress, wildfire exposure, and environmental degradation.

**Abstract:**

Deforestation promotes environmental changes capable of altering regional microclimatic dynamics, intensifying wildfires, and increasing population exposure to cardiovascular risk factors. This study investigated the spatiotemporal association between deforestation and acute myocardial infarction mortality across health regions of the Brazilian Amazon between 2000 and 2023. An ecological study design was adopted using data aggregated by health region and year. Generalized additive models with a negative binomial distribution were fitted to evaluate nonlinear associations between deforestation and acute myocardial infarction mortality, including temporal lag analyses of one, two, and three years. Spatial dynamics were further investigated through Bayesian spatiotemporal modeling incorporating structured spatial effects and a smoothed temporal trend. A significant nonlinear association was identified between deforestation and acute myocardial infarction mortality, with progressive risk intensification observed in areas subjected to greater environmental degradation. Lagged models demonstrated persistence of the association over time, suggesting cumulative effects of environmental exposure. Spatial analysis revealed an expansion of areas with elevated relative risk, particularly within the Arc of Deforestation of the Amazon region. Overall, the findings indicate that deforestation may act as an important socioenvironmental determinant of cardiovascular health in the Brazilian Amazon.

## 1. Introduction

Cardiovascular diseases remain the leading cause of morbidity and mortality worldwide, constituting a major and persistent challenge for healthcare systems [1]. This burden is particularly pronounced in low- and middle-income countries, where social inequalities, structural limitations, and adverse environmental conditions intensify cardiovascular risk determinants and exacerbate health disparities [2,3]. Among cardiovascular conditions, acute myocardial infarction stands out because of its high lethality, rapid clinical progression, and substantial contribution to premature mortality, remaining a major contributor to hospitalization and mortality worldwide [4]. Although advances in therapeutic strategies and expanded access to diagnostic and treatment services have improved clinical outcomes, the occurrence and severity of acute myocardial infarction continue to be strongly shaped by socioenvironmental determinants, especially in regions undergoing profound territorial transformation and marked structural vulnerability [5].

Historically, the epidemiological understanding of acute myocardial infarction has been predominantly focused on classical cardiovascular risk factors, including arterial hypertension, diabetes mellitus, dyslipidemia, obesity, smoking, and physical inactivity [6,7]. However, growing evidence indicates that environmental exposures also play a substantial role in cardiovascular pathophysiology, particularly those related to air pollution, rising temperatures, microclimatic instability, and ecosystem degradation [8,9,10]. These exposures may contribute to acute coronary events through complex biological pathways involving systemic inflammation, oxidative stress, endothelial dysfunction, sympathetic nervous system activation, and destabilization of atherosclerotic plaques [11]. Consequently, environmental degradation has increasingly been recognized as a relevant component in the multifactorial etiology of cardiovascular diseases.

Within this framework, deforestation emerges as an important indirect environmental determinant of cardiovascular health, particularly in tropical regions subjected to intense land-use conversion [5]. The removal of native vegetation profoundly disrupts local climatic regulation, promoting increased land surface temperatures, reduced relative humidity, heat island formation, intensification of wildfires, and deterioration of air quality [12,13,14,15]. These environmental changes extend beyond ecological imbalance, contributing to greater human exposure to cardiometabolic and cardiovascular risk factors, especially among socially vulnerable populations disproportionately affected by environmental degradation [16].

In the Brazilian Amazon, this process has gained relevance due to the rapid expansion of agricultural frontiers, mining activities, unplanned urbanization, and large-scale infrastructure projects, all of which have intensified forest fragmentation and persistently altered the environmental and social dynamics of the region [17,18]. In addition to biodiversity loss and its well-established impacts on infectious and zoonotic diseases, deforestation may also influence cardiovascular outcomes through cumulative and long-term modifications in thermal and atmospheric exposures [19]. Importantly, areas experiencing greater environmental degradation frequently overlap with regions characterized by limited healthcare coverage, heightened socioeconomic vulnerability, and reduced health system responsiveness, thereby potentially amplifying the burden of cardiovascular morbidity and mortality [2]. Despite these concerns, investigations examining deforestation as a spatiotemporal determinant of acute myocardial infarction mortality remain scarce, particularly in large tropical regions undergoing accelerated environmental transformation.

Understanding the relationship between deforestation and acute myocardial infarction has become increasingly relevant in the context of rapidly intensifying environmental change. The identification of spatial patterns and potential delayed effects associated with environmental degradation may contribute to territorial planning, strengthen epidemiological surveillance strategies, and support the development of integrated public policies linking environmental preservation and population health. In this context, the present study aimed to investigate the spatiotemporal association between deforestation and acute myocardial infarction mortality across health regions of the Brazilian Amazon between 2000 and 2023 using nonlinear inferential models and a Bayesian risk-mapping approach. We hypothesized that increasing deforestation would be associated with a progressive intensification of acute myocardial infarction risk, reflecting the cumulative impacts of environmental degradation on cardiovascular health at the population level.

## 2. Materials and Methods

### 2.1. Study Area

The study was conducted in the Brazilian Amazon, a macroregion encompassing approximately 5.2 million km^2^ and corresponding to nearly 61% of the Brazilian territory. This region comprises nine states, Acre, Amapá, Amazonas, Maranhão, Mato Grosso, Pará, Rondônia, Roraima, and Tocantins, and is characterized by pronounced socioenvironmental heterogeneity, with uneven population distribution across consolidated urban centers and extensive rural and peri-urban areas [17]. The total population is estimated to exceed 28 million inhabitants and is marked by substantial socioeconomic and structural inequalities [20].

Historically, the Amazon has undergone intense territorial transformation processes, particularly since the 1970s, driven by road network expansion and governmental policies promoting territorial occupation and agricultural development [21]. These processes have resulted in profound changes in land use and land cover, promoting forest fragmentation, expansion of deforested areas, and persistent alterations in regional microclimatic patterns. Such environmental modifications have been associated with heat island formation, rising land surface temperatures, and broader disruptions in local environmental dynamics.

### 2.2. Data Collection

Data on deaths from acute myocardial infarction and annual deforested area (km^2^) were collected and aggregated according to the health regions of the Brazilian Amazon for the period between 2000 and 2023. Health regions are territorial units adopted by the Brazilian Unified Health System (Sistema Único de Saúde—SUS) for healthcare organization, planning, and decentralization, grouping contiguous municipalities based on socioeconomic characteristics and the existence of integrated healthcare networks [21]. The use of these spatial units reduces statistical instability associated with small populations and mitigates the heterogeneity commonly observed in municipality-level analyses.

Data on deaths from acute myocardial infarction, confirmed according to clinical criteria and recorded in official health information systems, were obtained from DataSUS, the public platform maintained by the Brazilian Ministry of Health that consolidates notifications from national health surveillance systems [22]. Population estimates by year and health region were also retrieved from DataSUS and used to calculate annual mortality rates. Population data for census years were based on official demographic censuses, whereas intercensal estimates were provided by the Ministry of Health, ensuring temporal consistency throughout the study period. Mortality was selected as the primary outcome because death records are systematically collected and consistently available across all health regions and study years, providing a standardized indicator for long-term spatiotemporal analyses. Furthermore, acute myocardial infarction remains a major contributor to cardiovascular mortality worldwide, making mortality rates an appropriate population-level measure of cardiovascular burden in ecological studies.

Annual deforestation data (km^2^) were obtained from the Program for Monitoring Deforestation in the Legal Amazon by Satellite (PRODES), developed by the Brazilian National Institute for Space Research (Instituto Nacional de Pesquisas Espaciais—INPE). PRODES provides annual estimates of deforested areas based on remote sensing imagery, enabling systematic monitoring of land-use dynamics across the Amazon region. Because deforestation data were originally available at the municipal level, territorial correspondence procedures were applied to integrate them into health regions. The data were subsequently aggregated by year and region to ensure compatibility between administrative boundaries and the analytical units adopted in the study.

### 2.3. Data Analysis

All analyses were performed using GNU R software version 4.4.1 [23]. Acute myocardial infarction mortality was expressed as rates per 100,000 inhabitants, allowing standardization across regions with different population sizes. For temporal and spatial analyses, the study period was further grouped into three intervals: 2000–2007, 2008–2015, and 2016–2023.

The initial inferential analysis was conducted using generalized additive models (GAM) with a negative binomial distribution, which is appropriate for overdispersed count data. The outcome variable corresponded to the number of acute myocardial infarction deaths, while the logarithm of the population was included as an offset parameter to model mortality rates rather than crude counts. The main model incorporated a smoothed effect of deforestation and a nonlinear temporal trend. This approach enabled the assessment of nonlinear associations between deforestation and acute myocardial infarction mortality, as well as the identification of potential thresholds associated with progressive risk intensification along the environmental exposure gradient. Model adequacy was evaluated through residual diagnostics, including assessment of residual dispersion.

To investigate potential delayed effects of deforestation on acute myocardial infarction mortality, additional models incorporating temporal lags of one, two, and three years were constructed using lagged deforestation variables (desmat_lag1, desmat_lag2, and desmat_lag3). This strategy allowed the evaluation of cumulative and delayed responses associated with environmental changes over time. The lag periods were defined a priori to assess short- and medium-term delayed effects potentially arising from progressive environmental changes associated with deforestation, including alterations in thermal conditions, air quality, and other environmental stressors that may influence cardiovascular health beyond the year of exposure.

Spatial analysis was performed using Bayesian spatiotemporal modeling through the Integrated Nested Laplace Approximation (INLA) framework [24,25], adopting a negative binomial distribution to account for overdispersion in the data. The model incorporated three main components: (i) a nonlinear deforestation effect modeled through a second-order random walk structure (RW2) after discretization of the continuous deforestation variable into 20 quantile-based groups, which ensured a balanced distribution of observations across exposure levels while preserving sufficient resolution to capture potential nonlinear relationships; (ii) structured and unstructured spatial effects modeled using the reparameterized Besag–York–Mollié model (BYM2) [26], allowing adjustment for spatial autocorrelation among neighboring health regions; and (iii) a smoothed temporal trend modeled through a first-order random walk structure (RW1), capturing serial temporal dependence throughout the study period.

Spatial dependence was defined based on first-order Queen contiguity among health regions. A neighborhood matrix was constructed and subsequently converted into a graph structure compatible with the INLA framework. The expected number of deaths (E) was directly incorporated into the model, allowing the linear predictor to represent log(μ/E) = log(RR), where RR corresponds to the adjusted relative risk. Accordingly, relative risk estimates were obtained by exponentiating the linear predictor [RR = exp(η)]. Median relative risk estimates, corresponding 95% credible intervals (95% CrI), and posterior probabilities of excess risk [PP(RR > 1)] were extracted. Posterior probabilities were used to identify areas with stronger evidence of elevated risk. Regions were classified as high-risk clusters when presenting PP(RR > 1) ≥ 0.80 and as low-risk clusters when presenting PP(RR > 1) ≤ 0.20, whereas intermediate values were considered non-significant [27]. This approach enabled the identification of spatial patterns associated with progressive risk intensification across the three analyzed periods.

## 3. Results

**Figure 1:**
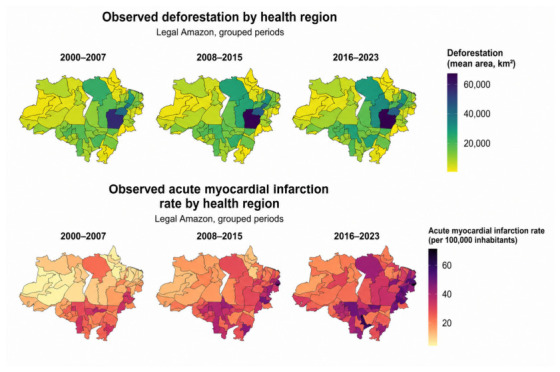
Observed deforestation and acute myocardial infarction mortality rates across health regions of the Brazilian Amazon during the periods 2000–2007, 2008–2015, and 2016–2023.

Summary statistics for annual deforestation area and acute myocardial infarction mortality rates across the three analytical periods are presented in Table 1. Both variables showed substantial temporal variability, with higher values observed during the most recent period. Visual inspection of the descriptive maps revealed a clear spatial and temporal correspondence between deforestation and acute myocardial infarction mortality across the Brazilian Amazon (Figure 1). Deforestation progressively intensified throughout the analyzed periods, with higher concentrations observed in central regions and within the so-called Arc of Deforestation, particularly across the southern, southeastern, and eastern portions of the Amazon. In the most recent period, expansion and consolidation of areas with greater deforestation magnitude became evident. Observed acute myocardial infarction mortality rates exhibited a similar spatial pattern, suggesting substantial geographic concordance with regions experiencing more intense deforestation.

**Figure 2:**
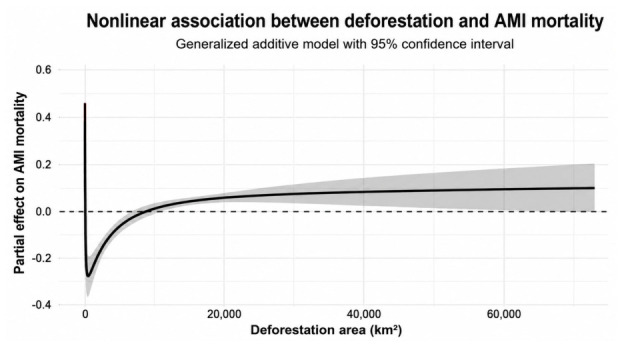
Smooth effect of deforestation on acute myocardial infarction mortality estimated by the generalized additive model. The solid line represents the estimated smooth function, the shaded area indicates the 95% confidence interval, and the dashed horizontal line represents the null effect. Deforestation was expressed as the annual deforested area (km^2^) aggregated by health region.

The generalized additive model with a negative binomial distribution further supported the association between deforestation and observed acute myocardial infarction mortality rates (β = 2.91; *p* < 0.001), with the smooth GAM function revealing a nonlinear exposure–response relationship characterized by progressively stronger effects as deforestation increased and a tendency toward stabilization at higher levels of exposure (Figure 2). The temporal component was also statistically significant (β = 2.25; *p* < 0.001), indicating consistent variation in mortality rates over time. Lagged analyses demonstrated persistence of the association between deforestation and acute myocardial infarction mortality across all temporal models. In the one-year lag model, the smoothed deforestation term remained statistically significant (β = 2.90; *p* < 0.001), as did the temporal component (β = 1.81; *p* < 0.001), with an adjusted R^2^ of 0.32. Similar findings were observed for the two-year lag model, in which both deforestation (β = 2.89; *p* < 0.001) and temporal trend (β = 1.78; *p* < 0.001) remained significant, also with an adjusted R^2^ of 0.32. In the three-year lag model, the smoothed deforestation effect continued to demonstrate statistical significance (β = 2.87; *p* < 0.001), together with the temporal component (β = 1.39; *p* < 0.001), yielding an adjusted R^2^ of 0.31.

Bayesian modeling demonstrated a progressive increase in the relative risk of acute myocardial infarction mortality across the analyzed periods. During the first period (2000–2007), the median relative risk was 0.66 (95% CrI: 0.61–0.82), accompanied by a low posterior probability of excess risk (PP[RR > 1] = 0.000008), indicating mortality levels below those expected. In the intermediate period (2008–2015), this pattern shifted toward a scenario of increased risk, with an estimated relative risk of 1.03 (95% CrI: 0.95–1.28) and a high posterior probability of excess risk (PP[RR > 1] = 0.89). Although the 95% confidence interval included the null value, the elevated posterior probability suggests a consistent trend toward higher acute myocardial infarction mortality relative to expected levels. This pattern became even more pronounced during the most recent period (2016–2023), when relative risk reached 1.42 (95% CrI: 1.35–1.81), also accompanied by the maximum posterior probability of excess risk (PP[RR > 1] = 1.00).

**Figure 3:**
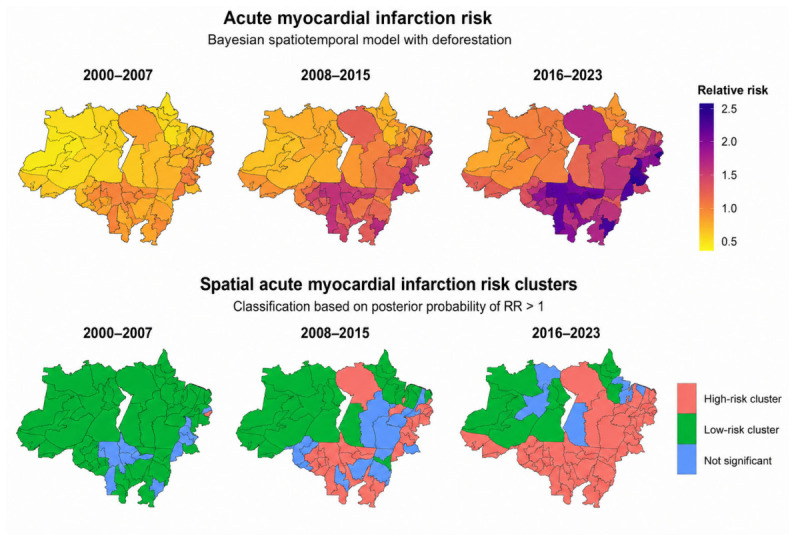
Adjusted relative risk and spatial risk clusters of acute myocardial infarction mortality obtained through Bayesian spatiotemporal modeling across health regions of the Brazilian Amazon during the periods 2000–2007, 2008–2015, and 2016–2023.

The Bayesian-derived maps (Figure 3) confirmed the progressive intensification of adjusted acute myocardial infarction risk over time. Between 2000 and 2007, low-risk regions predominated, with limited posterior probability of excess mortality. During the 2008–2015 period, a marked spatial expansion of risk areas was observed, indicating a transition toward a pattern of increased mortality, particularly in regions experiencing greater deforestation intensity. Between 2016 and 2023, this pattern became substantially more pronounced, with extensive areas being classified as high-risk clusters. These clusters were concentrated mainly in the eastern, southeastern, and southern portions of the Brazilian Amazon, spatially coinciding with regions historically characterized by elevated deforestation levels.

## 4. Discussion

The present study identified a significant association between deforestation and acute myocardial infarction mortality in the Brazilian Amazon, revealing a nonlinear pattern characterized by progressive risk intensification and persistent spatial clustering in areas exposed to greater environmental degradation. These findings suggest that the cardiovascular consequences associated with deforestation extend beyond localized ecological disruption, reflecting broader environmental transformations capable of substantially altering human exposure conditions. The expansion of deforested areas was consistently associated with the emergence of territories increasingly susceptible to acute myocardial infarction, particularly in regions marked by intense anthropogenic pressure and accelerated land-use conversion processes [17,28].

Forest loss profoundly disrupts local microclimatic regulation by increasing land surface temperatures, reducing evapotranspiration, decreasing relative humidity, and intensifying heat island formation [20]. These environmental alterations are frequently accompanied by increased wildfire activity and higher atmospheric particulate matter concentrations, thereby amplifying population exposure to pollutants associated with vascular inflammation and endothelial dysfunction [29]. Prolonged residence in thermally degraded environments may contribute to persistent sympathetic activation, elevated blood pressure, destabilization of atherosclerotic plaques, and increased myocardial metabolic demand, mechanisms consistently implicated in the pathophysiology of acute coronary syndromes, particularly among socially vulnerable populations [9,10,11]. Importantly, the nonlinear relationship identified in the present analysis indicates that these effects are not distributed uniformly across the exposure gradient, becoming substantially more pronounced after specific thresholds of territorial degradation are reached.

The persistence of the deforestation effect across lagged models further reinforces the cumulative nature of environmental exposure on cardiovascular health. Ecological changes resulting from forest loss may gradually alter thermal dynamics, atmospheric circulation, and air quality, generating chronic physiological stress processes that potentially manifest years after the onset of environmental degradation [19,30]. The cardiovascular response observed in this study likely reflects the sustained interaction between thermal stress, systemic inflammation, and the progressive aggravation of preexisting cardiometabolic conditions. The persistence of associations across the one-, two-, and three-year lag models is consistent with the expected temporal scale of these environmental and physiological processes [31]. This temporal pattern differs from that typically associated with acute environmental exposures, suggesting that deforestation may function as an important long-term territorial marker of cardiovascular risk [16,32]. The maintenance of statistically significant associations even after temporal lag strengthens the hypothesis that environmental degradation exerts persistent and cumulative effects on population cardiovascular health rather than merely transient impacts.

The progressive increase in acute myocardial infarction mortality over time suggests that the cardiovascular burden intensified alongside the environmental and territorial transformations observed across recent decades. Although part of this increase may be attributable to improvements in diagnostic capacity and broader access to healthcare services, the persistence of elevated spatial risks strongly indicates the contribution of environmental and socioeconomic determinants to the maintenance of this pattern [1,10]. Regions undergoing rapid agricultural expansion, mining activities, and unplanned urbanization frequently exhibit precarious sanitation infrastructure, marked social vulnerability, and limited healthcare system responsiveness, factors that may further increase population susceptibility to the environmental consequences associated with deforestation [2,33]. In this context, environmental degradation and structural inequalities likely interact synergistically, amplifying cardiovascular vulnerability in already marginalized territories.

High-risk spatial clusters were concentrated predominantly within the Arc of Deforestation of the Brazilian Amazon, a region historically characterized by intense forest fragmentation and sustained anthropogenic pressure [17]. This geographic overlap between environmental degradation and elevated cardiovascular risk suggests that deforestation does not operate as an isolated exposure, but rather as part of broader processes of territorial vulnerability and socioenvironmental destabilization. Municipalities located within these areas frequently experience persistent structural inequalities, limited access to specialized healthcare services, long distances to referral centers, and logistical barriers to emergency cardiovascular care [3]. Consequently, the coexistence of heightened environmental exposure and reduced healthcare capacity may contribute to disproportionately greater cardiovascular vulnerability and worse health outcomes at the regional level [2].

Comparable patterns have previously been reported in studies associating thermal extremes, air pollution, and wildfire exposure with increased hospitalizations and mortality due to acute myocardial infarction [19,34]. Nevertheless, most previous investigations have focused primarily on isolated and short-term environmental exposures, particularly in urban contexts [35]. The present analysis expands this perspective by demonstrating that large-scale structural environmental transformations, such as deforestation, may also influence the spatiotemporal dynamics of cardiovascular diseases over extensive territorial scales. This broader ecological approach contributes to advancing the understanding of how chronic environmental degradation may shape cardiovascular risk beyond traditionally investigated urban pollutants and climatic events.

The findings of this study carry important implications for public health and territorial planning in the Brazilian Amazon. Strategies aimed at controlling deforestation may generate indirect cardiovascular health benefits by reducing population exposure to thermal extremes, microclimatic degradation, and pollution associated with wildfires [12]. In this context, integrating environmental and epidemiological surveillance becomes particularly relevant in regions experiencing rapid land-use transformation. Monitoring systems capable of incorporating environmental indicators may facilitate the early identification of priority areas and strengthen preventive interventions targeting the most vulnerable populations. Moreover, the incorporation of environmental determinants into cardiovascular surveillance frameworks may contribute to more comprehensive and territorially sensitive public health strategies.

Despite the analytical robustness of the study, some limitations should be acknowledged. The ecological design precludes causal inference at the individual level and does not allow adjustment for important clinical determinants of cardiovascular disease, including hypertension, diabetes, smoking, obesity, and other individual risk factors. The use of secondary data may be affected by underreporting, variations in diagnostic capacity, and differences in data quality and healthcare access across regions. In addition, the models did not include area-level indicators such as socioeconomic conditions, healthcare infrastructure, urbanization, or the regional distribution of cardiovascular risk factors because standardized and temporally consistent data were not available for all health regions throughout the study period. As a result, these contextual characteristics may have contributed to the observed spatiotemporal patterns and could represent sources of residual confounding. Furthermore, the adjusted R^2^ values indicate that deforestation explained only part of the variability in acute myocardial infarction mortality, reflecting the inherently complex and multifactorial nature of cardiovascular disease. Therefore, the findings should be interpreted as evidence of a population-level ecological association rather than as proof of an independent causal effect of deforestation. Nevertheless, the extended temporal series, broad territorial coverage, and integration of nonlinear inferential modeling with Bayesian spatiotemporal analysis substantially strengthen the robustness and consistency of the findings.

## 5. Conclusions

The results demonstrate that deforestation was significantly associated with acute myocardial infarction mortality in the Brazilian Amazon, revealing a nonlinear pattern characterized by temporal persistence and progressive spatial intensification of risk in regions subjected to greater anthropogenic pressure. The observed association suggests that environmental transformations resulting from forest loss may operate as structural determinants of cardiovascular health, particularly in territories marked by socioeconomic vulnerability and limited healthcare capacity. The persistence of high-risk clusters within the Arc of Deforestation further reinforces the overlap between environmental degradation and health inequalities. Collectively, these findings expand the current understanding of the indirect impacts of deforestation on noncommunicable diseases and underscore the need to integrate environmental surveillance, territorial planning, and cardiovascular health policies into broader strategies aimed at protecting the Amazon region.

## Figures and Tables

**Table 1:** Descriptive statistics of annual deforestation area and acute myocardial infarction (AMI) mortality rates in the Brazilian Amazon. Values represent annual observations aggregated by health region and are presented as mean ± standard deviation (SD) and range (minimum–maximum).

Variable	Period	Mean ± SD	Range
Deforestation (km^2^)	2000–2007	9617 ± 9139	0.0–60,473
	2008–2015	11,244 ± 10,626	0.1–64,573
	2016–2023	12,214 ± 11,445	0.1–68,968
AMI mortality rate (100,000 inhabitants)	2000–2007	17.9 ± 11.2	0.0–68.5
	2008–2015	27.2 ± 14.6	0.0–98.7
	2016–2023	36.6 ± 22.8	3.8–234.5

## Data Availability

The data and R code associated with this manuscript are available on figshare: https://doi.org/10.6084/m9.figshare.32408400. The datasets used in this study are publicly available from DataSUS (https://datasus.saude.gov.br/) and the PRODES monitoring system (https://terrabrasilis.dpi.inpe.br/en/home-page/). All datasets were accessed in 1 January 2026.
